# Prevalence of posterior alveolar bony dehiscence and fenestration in adults with posterior crossbite: a CBCT study

**DOI:** 10.1186/s40510-020-00308-6

**Published:** 2020-03-16

**Authors:** Jin Young Choi, Kishore Chaudhry, Edwin Parks, Ji Hyun Ahn

**Affiliations:** 1 College of Dental Medicine, Advanced Education in Orthodontics and Dentofacial Orthopedics, Roseman University of Health Sciences, 4 Sunset Way, Bldg C, Henderson, NV 89014 USA; 2 Department of Oral Pathology, Medicine, and Radiology, Indiana University School of Dentistry, Indianapolis, USA

**Keywords:** Alveolar bony defect, Dehiscence, Fenestration, Posterior crossbite, CBCT, Adults, Prevalence

## Abstract

**Background:**

Correcting posterior crossbite in adult patients using nonsurgical methods may involve buccolingual tooth movement. Knowing the extent of the pretreatment alveolar bony dehiscences and fenestrations in the posterior area will aid orthodontists in planning posterior crossbite patients accordingly to minimize posttreatment bony defects. Before the advent of cone beam computed tomography (CBCT), observing buccal and lingual bony defects was not possible unless other treatment needs allowed for an open-flap procedure. With CBCT technology, we can now detect posterior defects with some accuracy. The aim of the present study was to determine the prevalence of posterior alveolar bony dehiscence and fenestration in adults with posterior crossbite compared with noncrossbite adults.

**Methods:**

The study group consisted of pretreatment CBCTs of 28 samples with at least one or more teeth in posterior crossbite or edgebite. The comparison group consisted of pretreatment CBCTs of 28 samples with no posterior crossbite or edgebite. All buccal and lingual sides of the upper and lower posterior segments were measured for the presence of dehiscence, fenestration, and combined total bony defects.

**Results:**

The prevalence of total bony defects was higher in the study group (61.6%) than in the comparison group (52.1%) (*p* < 0.05). While there was no difference in prevalence between crossbite teeth in the study group and noncrossbite teeth in the comparison group, the noncrossbite teeth in the study group showed a higher prevalence of total bony defects, dehiscence, and fenestration than the noncrossbite teeth in the comparison group (*p* < 0.05). The prevalence of dehiscence was higher in the study group (41.2%) than in the comparison group (33.3%) (*p* < 0.05). Neither the prevalence of fenestration nor the mean bony defect size showed statistical significance between the two groups. First premolars showed a higher prevalence of dehiscence than other posterior teeth, and maxillary posterior teeth had a higher prevalence of fenestration than mandibular posterior teeth. Among the maxillary posterior teeth, second premolars had the least amount of fenestration.

**Conclusions:**

Adult subjects with posterior crossbite had a higher prevalence of total bony defects and dehiscence, especially buccal dehiscence, in the posterior region than subjects with no posterior crossbite. This was due to the high prevalence observed in the noncrossbite teeth in posterior crossbite subjects.

## Background

Identifying alveolar bony fenestrations and dehiscences prior to orthodontic treatment is helpful to orthodontists for several reasons [1]. Studies have shown that the incidence of alveolar bony dehiscence and fenestration decreases the bony support of teeth, and in the presence of plaque-induced gingival inflammation, the lack of bony support during orthodontic movement can be detrimental to the health of the teeth and the periodontium [2]. An undetected and undiagnosed buccal alveolar bone defect poses greater potential for treatment relapse [3] and gingival recession, leading to unaesthetic finishing of orthodontic treatment and tooth sensitivity [4].

Detection of alveolar bony dehiscences and fenestrations was not possible with traditional 2D imaging. However, with the advent of cone beam computed tomography (CBCT), we now have the means to visualize these defects three dimensionally. Studies have examined alveolar bony defects using CBCT in children with cleft lip and palate [5], adolescents undergoing rapid maxillary expansion [6, 7], and adults with different vertical skeletal patterns [8, 9] and different skeletal malocclusions [10]. However, there has been no study examining alveolar bony defects in adults with posterior crossbite. When treating patients with posterior crossbite, the treatment modality may involve orthodontic tooth movement through a thin osseous plate, which may lead to bony defects [11], and having a good pretreatment assessment of existing alveolar bony defects becomes a crucial diagnostic step [12] in treating such cases. It is recommended that orthodontists know the anatomical limits of tooth movement to be aware of potential periodontal problems that can worsen during orthodontic treatment [12].

Studies have investigated the accuracy and limitations of CBCT as a detection tool for alveolar bony defects in vivo and in vitro. CBCT has a high specificity and a high negative predictive value for both dehiscence and fenestration but a low positive predictive value, especially for fenestration [1, 13]. This means that CBCT can overestimate bony defects, especially fenestration. However, in terms of orthodontists using bony defect data as a precaution prior to treatment, it can be viewed that overestimation adds to the side of caution rather than giving misinformation. As long as users of CBCT understand the extent of its accuracy, clinicians can still use the bony defect information within the boundaries of the overestimation limitation.

The primary aim of this study was to determine the prevalence of posterior alveolar bony dehiscence and fenestration in adults with posterior crossbite and compare the prevalence to that of noncrossbite adults. The secondary aim of this study was to evaluate whether there is a difference in the prevalence of alveolar bony defects between teeth in crossbite and teeth not in crossbite.

Crossbite teeth included both teeth in full crossbite and teeth in edgebite since both are not in a normal buccal overjet condition. The results excluding teeth in the edgebite are provided as supplementary information in the “Appendix” section to show the effect of only full crossbite teeth.

## Methods

### Sample selection

This project was approved by the Institutional Review Board of Roseman University of Health Sciences (1187542-1). Informed consent to participate in research studies at Roseman University of Health Sciences was obtained from all patients at the time of the pretreatment CBCT scan. CBCT images were taken with iCAT (Model 17-19; Imaging Sciences International, Hatfield, PA, USA) with 0.4-mm voxel size, 16 × 13 cm field of view (FOV), 120 kVp, 5 mA, and 17.5 s exposure time until February 28, 2013. Then, the setting was changed to 0.3-mm voxel size, 23 × 17 cm FOV, 120 kVp, 5 mA, and 17.5 s exposure time. The change in CBCT setting was implemented by the Orthodontic Clinic at Roseman University of Health Sciences to increase resolution with reduced voxel size and to capture more pertinent structures with a larger FOV.

In this retrospective study, adult subjects aged 18 to 35 years old were selected. A total of 453 subjects were identified who were in this age group at the time of the pretreatment CBCT record at Roseman Orthodontic Clinic in Henderson, Nevada, from March 2009 to May 2018. The inclusion criteria for the study group were as follows: (1) the presence of posterior crossbite or edgebite on at least one tooth, (2) permanent dentition without missing posterior teeth except third molars, (3) up to 2 mm of crowding per posterior quadrant, and (4) the presence of pretreatment CBCT image files, intraoral photos, and scanned dental cast images. Posterior crossbite in this study was defined as having lingual crossbite [14] or edgebite on at least one of the posterior teeth, from the first premolar to the second molar.

The exclusion criteria for the study group were as follows: subjects with (1) obvious pathology (cyst or tumor), (2) posterior open bite, (3) multiple carious lesions, restoration, abfraction, or abrasion in the near-cervical area, (4) signs of periodontitis, (5) skeletal dysplasia and severe skeletal discrepancy that did not cause functional occlusion, (6) history of orthodontic treatment, and (7) the presence of systemic disease that may affect the bony condition (i.e., thyroid disease, osteonecrosis of the jaw, etc.), (8) treatment with medications that may affect the bony condition (i.e., bisphosphonates, RANKL inhibitors, etc.).

After applying the inclusion and exclusion criteria, the study group consisting of 28 adult subjects, 12 males and 16 females, was established.

For the comparison group, the age, gender, and mandibular plane angle (SN-GoMe) were matched to those of the study group. The inclusion criteria for the comparison group were as follows: (1) the absence of posterior crossbite or edgebite, (2) normal dentoalveolar transverse width in the posterior aspect, (3) permanent dentition without missing posterior teeth except third molars, (4) up to 2 mm of crowding per posterior quadrant, and (5) the presence of pretreatment CBCT image files, intraoral photos, and scanned dental cast images. Normal dentoalveolar transverse width in the posterior region was determined by the ABO standard for proper overjet. Buccal cusp tips of the mandibular posterior teeth had contact in the center of the occlusal surfaces, buccolingually, of the maxillary posterior teeth. The exclusion criteria for the comparison group were the same as those of the study group.

The power analysis was performed using a sample size from a previous study on the prevalence of alveolar bony defects [8]. With a power of 80% and confidence level of 95%, the power analysis determined that 23 samples were needed for each group. Thus, the 56 total samples, 28 in each group, were adequate. Twelve teeth were omitted before the analysis phase: one tooth was a deciduous molar with a congenitally missing permanent successor, four teeth were in buccal crossbite, and seven teeth had a metal crown margin near the cementoenamel junction (CEJ) area. Thus, 884 posterior teeth were included: 437 teeth in the study group and 447 teeth in the comparison group. The demographic information of the subjects is presented in Table 1.

**Table 1:** Demographic data of subjects

	Total	Crossbite group	Noncrossbite group	*p* value
Number of subjects (M, F)	56 (24,32)	28 (12, 16)	28 (12, 16)	N/A
Number of teeth (M, F)	884 (379, 505)	437 (187, 250)	447 (192, 255)	N/A
CBCT setting	Old 18 (32.1%) New 38 (67.9%)	Old 11 (39.3%) New 17 (60.7%)	Old 7 (25%) New 21 (75%)	N/A
Mean age ± SD (years)	23.6 ± 5.3	23.57 ± 5.2	23.75 ± 5.5	0.901
SN-MP angle ± SD (°)	34.46 ± 6.02	34.49 ± 6.09	34.43 ± 6.05	0.970
ANB (°)	3.11 ± 2.46	2.33 ± 2.08	3.89 ± 2.60	0.016*

(*p* value is between crossbite group and noncrossbite group.)

*p < 0.05 statistically significant

Table 2 demonstrates the distribution of vertical facial types, such as hypodivergent, normodivergent, and hyperdivergent, in each group and compares the mean SN-MP angles of each vertical facial type between the crossbite and noncrossbite groups. The two groups are well matched, as seen in the similarity of the distribution and the mean SN-MP of each facial type. More than half of the samples were normodivergent, followed by hyperdivergent subjects. Hypodivergent subjects were only two in each group (7.1%).

**Table 2:** Vertical facial type comparison between the crossbite and noncrossbite groups

		Crossbite group	Noncrossbite group	*p* value
Hypodivergent (SN-MP ≤ 26)	Number of subjects	2 (7.1%)	2 (7.1%)	
	Mean SN-MP	22.95 ± 1.34	23.85 ± 2.33	0.683
Normodivergent (26 <SN-MP< 38)	Number of subjects	16 (57.1%)	16 (57.1%)	
	Mean SN-MP	32.10 ± 4.04	31.58 ± 2.87	0.525
Hyperdivergent (SN-MP ≥ 38)	Number of subjects	10 (35.7%)	10 (35.7%)	
	Mean SN-MP	40.60 ± 1.65	41.09 ± 2.65	0.626
Total subjects	Number of subjects	28 (100%)	28 (100%)	
	Mean SN-MP	34.49 ± 6.09	34.43 ± 6.05	0.970

For the purpose of examining bony defect prevalence by crossbite status of individual teeth, teeth in the study group were further divided into two subgroups, and teeth in the comparison group were assigned to its own subgroup (Table 3). Subgroup 1 (SG1) consisted of crossbite teeth in the study group, subgroup 2 (SG2) consisted of noncrossbite teeth in the study group, and subgroup 3 (SG3) consisted of noncrossbite (that is, all) teeth in the comparison group (Table 3).

**Table 3:** Distribution of the subgroups (SGs)

	Teeth in crossbite (no. of teeth)	Teeth not in crossbite (no. of teeth)	Total
Crossbite group	Subgroup 1 (110) Full crossbite (89) Edgebite (21)	Subgroup 2 (327)	(437)
Noncrossbite group	(0)	Subgroup 3 (447)	(447)
Total	(110)	(774)	(884)

### Blinding/anonymizing

All CBCT DICOM files were anonymized by removing all patient identifiable information, and then randomized computer-generated case numbers (1 through 56) were assigned for the blind test. The investigator, J.C., did not have access to the subject-specific demographic information of the CBCT DICOM files until all bony defect measurements were completed.

### Bony defect measurement method

**Fig. 1:**
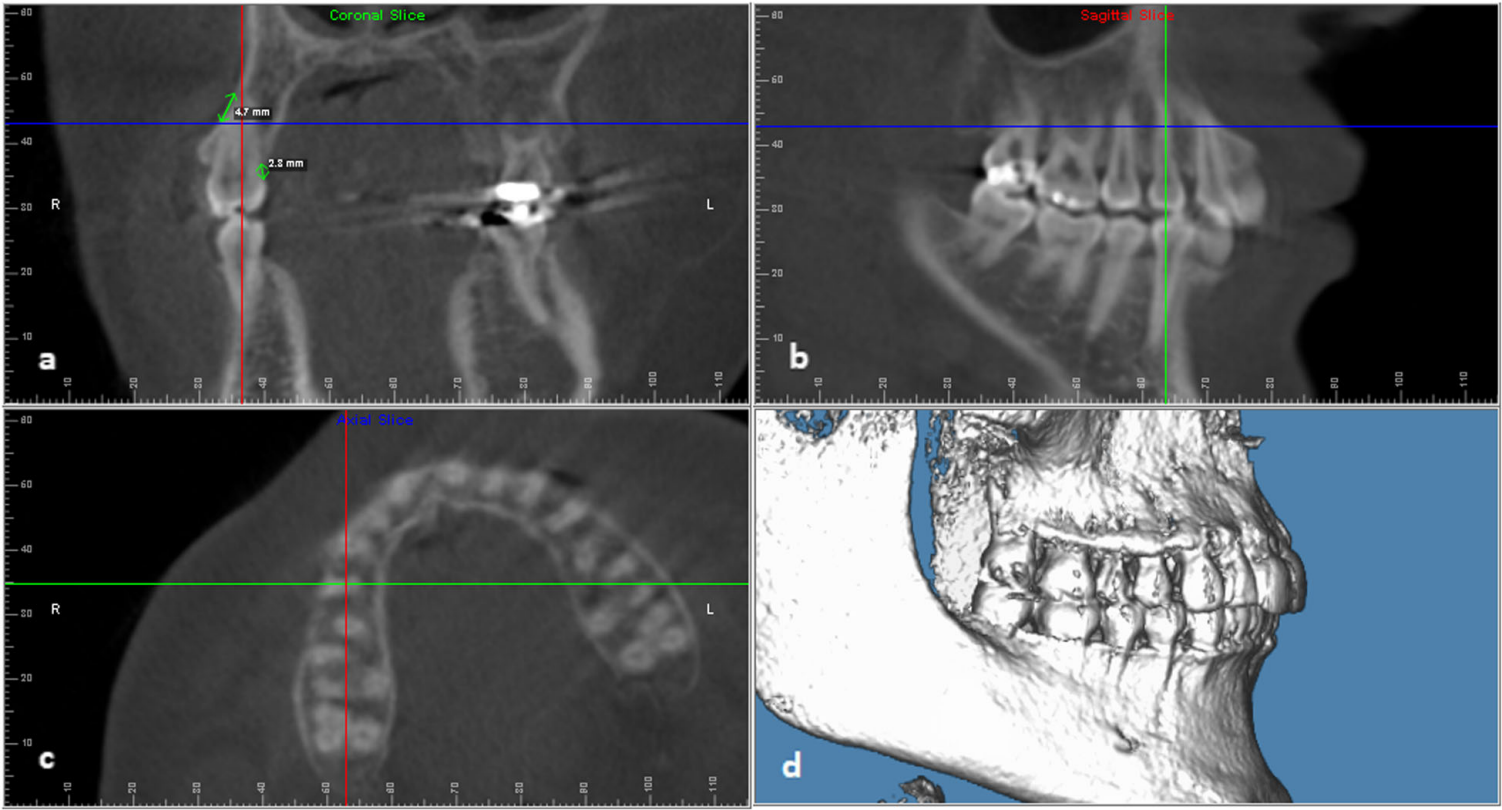
Bony defect measurement method. Orientation of coronal, axial, and sagittal slices on the multiplanar view and measurement of buccal bony fenestration and lingual dehiscence on UR4 on the coronal cross-section. (**a** upper left: coronal view, **b** upper right: sagittal view, **c** lower left: axial view, **d** lower right: volume rendered image)

All CBCT images were viewed and measured using the Dolphin Imaging 11.8 Premium software by the same investigator (J.C.). The image was oriented using the Frankfort-Horizontal (FH) line so that the FH plane is parallel to the floor, and the midsagittal plane is perpendicular to the FH plane. Each posterior quadrant was viewed in the multiplanar view (coronal, axial, sagittal panels) with 3 times magnification. Once enlarged, each quadrant was reoriented so that the posterior occlusal plane was level on the sagittal view, and the posterior segment was lined up anteroposteriorly on the axial view. If a certain tooth had a significant tipping or angulation, each cross-sectional slice’s orientation was readjusted to have the sagittal section parallel to the long axis of the tooth and the axial slice perpendicular to the long axis of the tooth before detecting bony defects (Fig. 1).

For the lesion to be counted as a dehiscence, it had to be equal to or larger than 2 mm in its vertical distance from the CEJ. This requirement was aimed to eliminate counting normal alveolar bone level as a dehiscence, which is usually 1.5 to 2.0 mm below the level of the CEJ [15]. There was no minimum required lesion size for fenestration. If any amount of bone was denuded on the root surface but not continuous to marginal bone [11], it was counted and measured as a fenestration. Sun et al. [13] found 2.2 mm as a critical point for both dehiscence and fenestration measurements with an improved Youden Index. However, the increase in the Youden Index was not significant. In this study, 2.0 mm or higher was used for dehiscence measurements and 0.0 mm or higher was used for fenestration according to the definitions of dehiscence and fenestration and was consistent with previous studies on bony defect measurements [6, 9, 10, 12].

Once the defect was confirmed in 3-sequential views [13] in both coronal and axial sections, the vertical diameter of the bony defect was measured on coronal sections up to one decimal point using the 2D line tool on Dolphin Imaging.

### Statistical analysis

All statistical analyses were performed with the Statistical Package for the Social Sciences (version 25.0; SPSS, Chicago, IL). Intraexaminer reliability was assessed by calculating the intraclass correlation coefficient (ICC) of 10% of randomly selected samples measured 1 week apart.

The chi-square test was used to compare the prevalence of alveolar bony defects between the crossbite group and noncrossbite group and to compare the prevalence of alveolar bony defects among the three subgroups.

Independent samples *T* tests were performed to compare the mean size of alveolar bony defects between the crossbite group and noncrossbite group. One-way ANOVA with a post hoc test was used to compare the mean size of alveolar bony defects among the three subgroups.

Pearson correlation analysis was used to examine the correlation between buccal dehiscence and lingual fenestration and between lingual dehiscence and buccal fenestration.

## Results

The ICC values for dehiscence and fenestration were 0.878 and 0.958, respectively, indicating that the measurements had good reliability.

A tooth was counted as the tooth with a bony defect when there was a bony defect on one side, either the buccal or lingual, or defects on both sides, as listed in Tables 4 and 5. Comparing the prevalence of total bony defects (the presence of dehiscence and/or fenestration) between posterior crossbite subjects and noncrossbite subjects (Table 4) revealed that it was significantly higher (9.5%, *p* = 0.005) in the crossbite group (61.6%) than in the noncrossbite group (52.1%). The prevalence of dehiscence was significantly higher (7.9%, *p* = 0.016) in the crossbite group (41.2%) than in the noncrossbite group (33.3%). The mean size of dehiscence was 0.5 mm larger in the study group, which was not enough to show a statistically significant difference (*p* = 0.058). The prevalence and mean defect size of fenestration in the study group were slightly higher than those in the comparison group, but the differences were not statistically significant.

**Table 4:** Prevalence and mean size of total bony defects, dehiscence, and fenestration in the study group (crossbite group) and comparison group (noncrossbite group)

		Crossbite group (*n* = 437) teeth	Noncrossbite group (*n* = 447) teeth	*p* value
Total bony defect prevalence (number of observations)		61.6% (269)	52.1% (233)	0.005**
Dehiscence	Prevalence (number of observations)	41.2% (180)	33.3% (149)	0.016*
	Mean size (mm) ± SD	4.47 ± 2.61	3.96 ± 2.26	0.058
Fenestration	Prevalence (number of observations)	34.6% (151)	28.6% (128)	0.058
	Mean size (mm) ± SD	4.37 ± 2.15	4.13 ± 1.83	0.305

*p < 0.05 statistically significant

**p < 0.01 statistically highly significant

**Table 5:** Prevalence and mean size of total bony defects, dehiscence, and fenestration by subgroup

		Crossbite group (*n* = 437)		Noncrossbite group (*n* = 447)	*p* value (SG1–SG2)	*p* value (SG1–SG3)	*p* value (SG2–SG3)
		SG1: teeth in crossbite (*n* = 110)	SG2: teeth not in crossbite (*n* = 327)	SG3: teeth not in crossbite (*n* = 447)			
Total bony defect prevalence (number of observations)		56.3% (62)	63.3% (207)	52.1% (233)	0.196	0.425	0.002**
Dehiscence	Prevalence (number of observations)	37.3% (41)	42.5% (139)	33.3% (149)	0.335	0.435	0.009**
	Mean size (mm) ± SD	4.28 ± 2.86	4.53 ± 2.55	3.96 ± 2.26	0.150		
Fenestration	Prevalence (number of observations)	30.9% (34)	35.8% (117)	28.6% (128)	0.353	0.638	0.035*
	Mean size (mm) ± SD	4.23 ± 2.14	4.42 ± 2.17	4.13 ± 1.83	0.525		

(Chi-square test for the prevalence comparison, ANOVA for the mean size comparison)

*p < 0.05 statistically significant (between subgroup 2 and subgroup 3)

**p < 0.01 statistically highly significant (between subgroup 2 and subgroup 3)

When the study group was divided by subgroup (Table 5) based on crossbite status, teeth in crossbite (subgroup 1, SG1) did not show any statistically significant difference in the prevalence or the mean size of bony defects from teeth not in crossbite in the comparison group (subgroup 3, SG3). In contrast, teeth not in crossbite in the study group (subgroup 2, SG2) showed a higher prevalence of total bony defects, dehiscence, and fenestration with larger defects than teeth in subgroup 3 or subgroup 1, which were fewer than 10%. There was a trend in which the bony defect prevalence and size were the highest in subgroup 2, followed by subgroup 1 and subgroup 3. The mean defect size difference for dehiscence and fenestration was only 0.3–0.6 mm.

An examination of the bony defect prevalence per buccal or lingual surface between the two groups revealed that the crossbite group (study group) had a 10% greater prevalence of buccal dehiscence than the noncrossbite group (comparison group) (Table 6), which was mainly due to the higher prevalence in subgroup 2 (Table 7). The lingual fenestration in subgroup 2 was also significantly higher than that in subgroups 1 or 3. The rest of the defects (i.e., lingual dehiscence, buccal fenestration, and mean size) did not show significant differences among subgroups.

**Table 6:** Dehiscence and fenestration prevalence and mean size by buccal and lingual surfaces in the crossbite group (study group) and the noncrossbite group (comparison group)

		Crossbite group (*n* = 437)	Noncrossbite group (*n* = 447)	*p* value
Buccal dehiscence	Prevalence (number of observations)	32.3% (141)	22.4% (100)	0.001**
	Mean size (mm) ± SD	4.51 ± 2.56	4.27 ± 2.54	0.473
Lingual dehiscence	Prevalence (number of observations)	25.9% (113)	21.35% (95)	0.107
	Mean size (mm) ± SD	3.24 ± 2.04	2.91 ± 1.13	0.150
Buccal fenestration	Prevalence (number of observations)	25.6% (112)	22.8% (102)	0.329
	Mean size (mm) ± SD	4.23 ± 1.87	4.15 ± 1.76	0.729
Lingual fenestration	Prevalence (number of observations)	11.7% (51)	7.8% (35)	0.054
	Mean size (mm) ± SD	4.47 ± 2.56	3.83 ± 1.96	0.220

**p < 0.01 statistically highly significant

**Table 7:** Dehiscence and fenestration prevalence and mean size by buccal and lingual surfaces by subgroup

		Crossbite group (*n* = 437)		Noncrossbite group (*n* = 447)	*p* value (SG1–SG2)	*p* value (SG1–SG3)	*p* value (SG2–SG3)
		SG1: teeth in crossbite (110)	SG2: teeth not in crossbite (327)	SG3: teeth not in crossbite (447)			
Buccal Dehiscence	Prevalence (number of observations)	27.3% (30)	33.9% (111)	22.4% (100)	0.195	0.276	< 0.001**
	Mean size (mm) ± SD	4.33 ± 2.87	4.56 ± 2.48	4.27 ± 2.54	0.703		
Lingual dehiscence	Prevalence (number of observations)	28.2% (31)	25.1% (82)	21.3% (95)	0.520	0.120	0.211
	Mean size (mm) ± SD	3.36 ± 2.38	3.19 ± 1.92	2.91 ± 1.13	0.351		
Buccal fenestration	Prevalence (number of observations)	26.4% (29)	25.4% (83)	22.8% (102)	0.838	0.432	0.409
	Mean size (mm) ± SD	3.69 ± 1.36	4.42 ± 1.99	4.15 ± 1.76	0.168		
Lingual fenestration	Prevalence (number of observations)	10% (11)	12.2% (40)	7.8% (35)	0.528	0.459	0.041*
	Mean size (mm) ± SD	5.42 ± 2.87	4.21 ± 2.45	3.83 ± 1.96	0.149		

(Chi-square test for the prevalence comparison, ANOVA for the mean size comparison)

*p < 0.05 statistically significant (between subgroup 2 and subgroup 3)

**p < 0.01 statistically highly significant (between subgroup 2 and subgroup 3)

Table 8 demonstrates the laterality of the prevalence of dehiscence and fenestration per crossbite and noncrossbite group. Some teeth had bony defects on the unilateral side, either buccal or lingual; some, on both sides. The buccal total is the combined prevalence of the buccal side only and both sides. The same method was applied for the lingual total, combining the prevalence of the lingual side only and both sides. Comparison of the bony defect prevalence between the buccal total and the lingual total revealed that the crossbite group and subgroup 2 had more bony dehiscences on the buccal side than on the lingual side. Subgroups 1 and 3 showed an even distribution of dehiscence prevalence on the buccal and lingual sides. In contrast, there was a significantly higher prevalence of fenestration on the buccal side than on the lingual side in all groups (Table 8).

**Table 8:** Comparison of the bony defect prevalence between the buccal and lingual sides

			Buccal only	Lingual only	Both sides	Buccal total	Lingual total	*p* value
Dehiscence prevalence (number of observations)	Crossbite group (*n* = 437)		15.3% (67)	8.9% (39)	16.9% (74)	32.3% (141)	25.9% (113)	0.037*
		SG 1 (*n* = 110)	9.1% (10)	10% (11)	18.2% (20)	27.3% (30)	28.2% (31)	0.880
		SG 2 (*n* = 327)	17.4% (57)	8.6% (28)	16.5% (54)	33.9% (111)	25.1% (82)	0.013*
	Noncrossbite group (*n* = 447)	Subgroup 3 (*n* = 447)	12.1% (54)	11% (49)	10.3% (46)	22.4% (100)	21.3% (95)	0.686
Fenestration prevalence (number of observations)	Crossbite group (*n* = 437)		22.7% (99)	8.7% (38)	3% (13)	25.6% (112)	11.7% (51)	< 0.001**
		SG 1 (*n* = 110)	20.9% (23)	4.5% (5)	5.5% (6)	26.4% (29)	10% (11)	0.002**
		SG 2 (*n* = 327)	23.2% (76)	10.1% (33)	2.1% (7)	25.4% (83)	12.2% (40)	< 0.001**
	Noncrossbite group (*n* = 447)	SG 3 (*n* = 447)	20.8% (93)	5.8% (26)	2.0% (9)	22.8% (102)	7.8% (35)	< 0.001**

(*p* value is between the buccal total prevalence and lingual total prevalence)

*p < 0.05 statistically significant

**p < 0.01 statistically highly significant

**Fig. 2:**
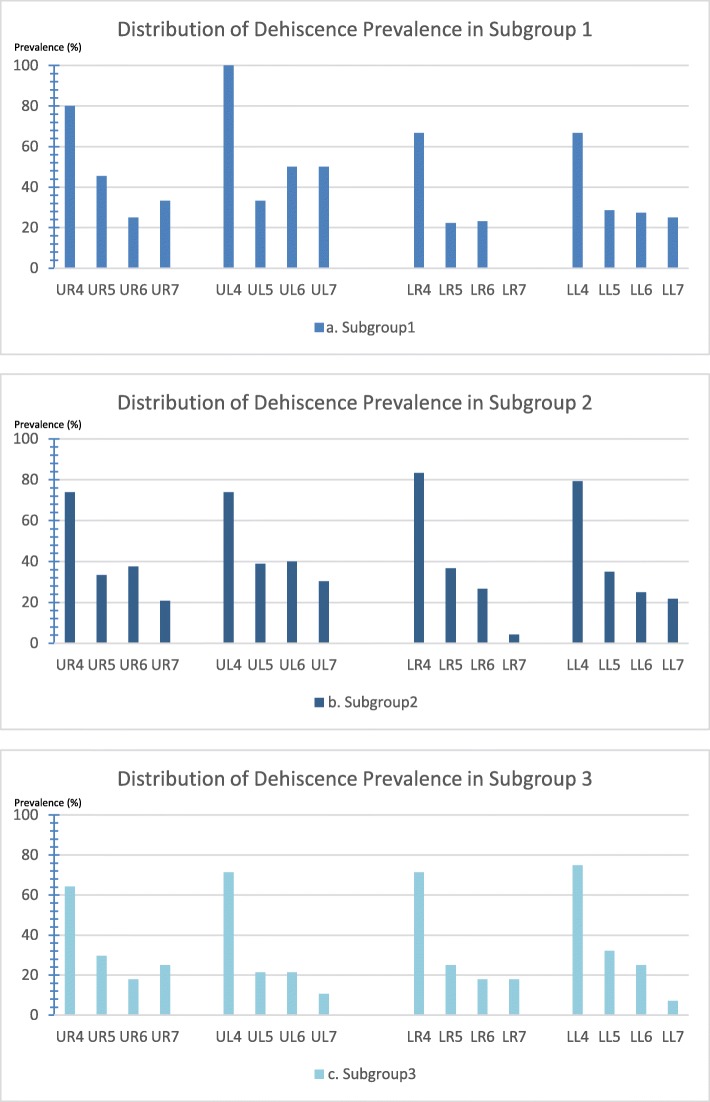
Distribution of the dehiscence prevalence among the posterior teeth by subgroup. **a** Subgroup 1, the crossbite teeth in the study group. **b** Subgroup 2, the noncrossbite teeth in the study group. **c** Subgroup 3, the noncrossbite teeth in the comparison group

**Fig. 3:**
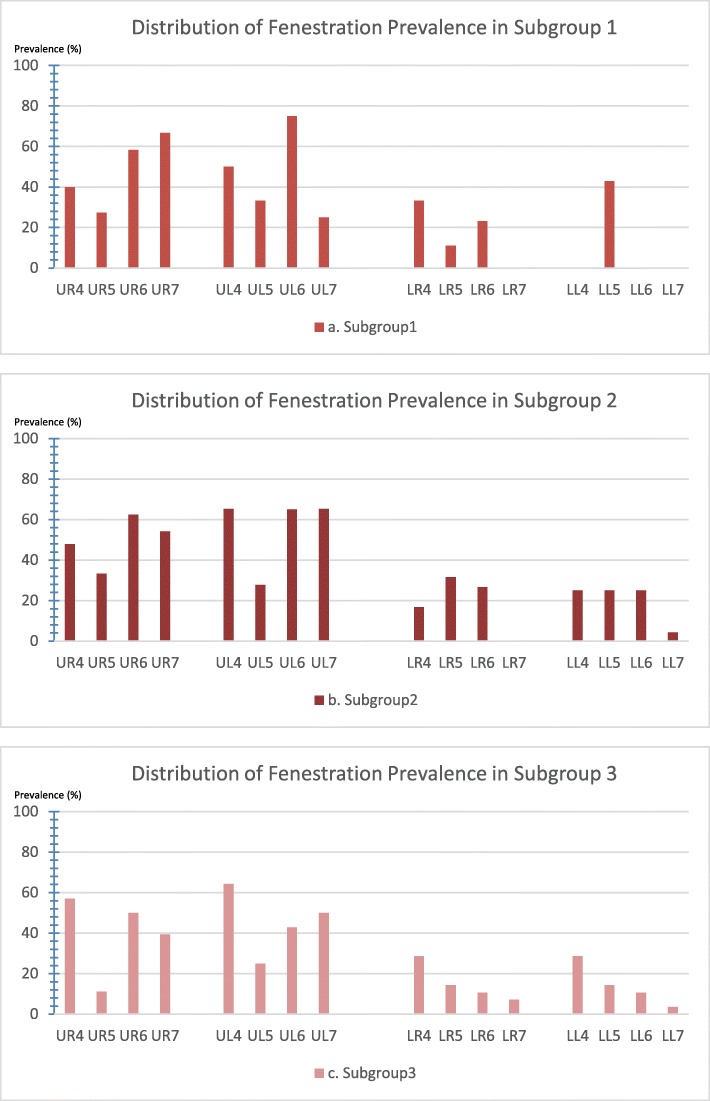
Distribution of the fenestration prevalence among the posterior teeth by subgroup. **a** Subgroup 1, the crossbite teeth in the study group. **b** Subgroup 2, the noncrossbite teeth in the study group. **c** Subgroup 3, the noncrossbite teeth in the comparison group

Figure 2 shows the distribution of dehiscence prevalence in each subgroup by posterior tooth type. Compared with other posterior teeth, the first premolars had the highest dehiscence prevalence among all subgroups. Figure 3 shows that fenestrations were more prevalent in maxillary teeth than in mandibular teeth in all subgroups. Within the maxilla, the second premolars had the lowest prevalence of fenestration.

There was no correlation between buccal dehiscence prevalence and lingual fenestration prevalence (− 0.047) or between lingual dehiscence and buccal fenestration (0.147).

## Discussion

Due to significantly higher ionizing radiation, CBCT should not be considered routine in orthodontics. Rather, the selection of CBCT imaging should be carefully made based on the initial clinical evaluation and should be justified based on individual need. The benefits of CBCT must outweigh the radiation risks, and per the ALARA principle, the size of the FOV must be carefully selected for each case [16]. Even though conventional radiographs, such as lateral cephalogram and panoramic X-ray, can provide necessary diagnostic information, there are cases where CBCT can provide more useful information, which can improve clinical confidence and efficacy: impacted canines, unerupted teeth, supernumerary teeth, severe root resorption, and severe skeletal discrepancies [16–18]. One of the useful pieces of information that can be obtained from CBCT, which was not possible with two-dimensional radiographic imaging, is the presence of alveolar bony defects in the posterior area. Being aware of existing bony defects in the posterior area becomes important, especially in cases where substantial buccolingual tooth movement or tipping is anticipated during orthodontic treatment, such as in posterior crossbite cases. Depending on which direction teeth are moved, existing bony defects can be either improved or worsened [19]. Improving bony defects can increase the stability of teeth, whereas worsening pre-existing bony defect conditions can lead to instability of teeth and periodontal problems [20, 21].

Regarding patient selection, growing patients (age under 18) were not included in the study, as previous studies showed that hormonal and functional changes associated with age influence cortical bone thickness [22, 23]. Subjects over age 35 were excluded from the study because of the increased risk of periodontitis in this age group [24], in order to eliminate introducing bone loss from periodontitis as a confounding factor.

The prevalence of total bony defects and dehiscence showed statistically significant differences between posterior crossbite subjects and noncrossbite subjects and among the three subgroups; however, the differences in prevalence were approximately 10%, which may not be a clinically meaningful difference.

Not all prevalence and mean defect size measurements had statistically significant differences among the three subgroups; however, they all followed a similar trend. The highest prevalence or largest size of defects was observed in the noncrossbite teeth in the crossbite group (SG2). The second highest was in the crossbite teeth in the crossbite group (SG1), and the lowest was in the noncrossbite group, which consisted of noncrossbite teeth only (SG3). This trend can be explained by considering the buccolingual inclination of teeth in dental compensation. Within posterior crossbite subjects, the teeth that are not in crossbite are under dental compensation, whereas the teeth in crossbite maintain their position within the bony housing [6, 7]. Thus, noncrossbite teeth in crossbite subjects had a higher prevalence of bony defects because they were positioned out of the bony housing in an effort to dentally compensate for the occlusal relationships. A follow-up study combined with buccolingual angular measurement of teeth is necessary to strengthen the interpretation.

The buccal side showed a higher prevalence of dehiscence and fenestration compared with the lingual side in our study, in agreement with a previous study [12]. The buccal side of bone tends to be thinner than the lingual or palatal side; thus, more buccal defects are anticipated. However, this prevalence could have been overestimated because a very thin layer of bone covering the buccal side can be missed easily and be counted as defects [1].

The first premolars showed a higher prevalence of dehiscence among posterior teeth in all subgroups. This is due to the relatively narrow alveolar bone width in this area compared with other posterior regions because the alveolar bone width narrows as it goes from posterior to anterior in both the maxilla and mandible. For the maxillary first premolar, it is also because of the two diverging prominent root morphologies, making this area more prone to developing bony defects.

The maxilla showed a higher prevalence of fenestration than the mandible. Previous studies [8–10, 12] have found similar results because the maxillary bone narrows from the cervical to apical level of teeth, and the mandible has a thicker cortical bone. Within the maxilla, there was less fenestration, especially on the second premolars compared with other maxillary posterior teeth. This is because maxillary second premolars are the only single rooted maxillary posterior teeth and do not have the diverging root prominence that comes from multirooted teeth.

As previous studies have shown, CBCT can overestimate bony defects because of its low positive predictive value, especially for fenestration [1, 13]. The values reported in this study for the prevalence of bony defects are most likely to be overestimated and contain some false-positive values. Thus, it is necessary to reinterpret the percentage of bony defects considering the chances of false-positive readings.

Important confounding factors for bony defect detection within and between subjects are alveolar bone thickness and periodontal biotype. Measurements of the alveolar bone thickness or clinical examination of the periodontal biotype were not conducted in this study; however, the study used vertical facial type data instead. According to Gracco et al. [25], vertical facial type is statistically significantly correlated with alveolar bone thickness. They found a greater alveolar bone thickness in hypodivergent patients than in hyperdivergent patients, although the study was limited to upper incisors only. A later study also found that hyperdivergent and normodivergent facial types have a higher prevalence of dehiscence than hypodivergent facial type [9]. Thus, the current literature indicates that hyper- and normodivergent facial types can be more prone to bony defects than hypodivergent facial type. Our study had a majority of hyper- and normodivergent facial types, which could have inflated the prevalence. However, the findings regarding the difference between the two groups (crossbite vs. noncrossbite) should not have been affected by the inflating effect of possible confounding factors because the distribution of facial types was matched between the two groups.

The study included edge-to-edge relationships in the crossbite teeth group. The supplementary information in the appendix section shows a separate analysis excluding the teeth in the edgebite. The results were similar except for an additional statistically significant difference found in the mean size of dehiscence and in the prevalence of fenestration between the crossbite group and noncrossbite group (Table 9 in the Appendix) and the dehiscence prevalence of the crossbite group between the buccal and lingual sides (Table 13 in the Appendix). However, a direct comparison of results based on the inclusion or exclusion of edgebite teeth may not be proper due to the small sample size of teeth in edgebite.

A correlation analysis was conducted to test whether buccal dehiscence occurred concurrently with lingual fenestration, and lingual dehiscence with buccal fenestration. No correlation was found in this investigation, but further study is needed to look at the relationship between the buccolingual inclination of teeth and the type and prevalence of bony defects.

### Limitation

The CBCT images used in this study were taken with two different settings as mentioned in the methods section. This is a limitation of a retrospective study because the investigator could not control the settings of CBCT images taken in the past. However, there is a study that showed that different CBCT settings may not have a major influence on the measurements of alveolar bone [26].

The limitation found in the selection of the sample was not having an exactly matching anterior-posterior skeletal pattern (ANB value) between the study group and comparison group, even though the difference was clinically acceptable (difference of 1.56°).

Dehiscence and fenestration are not symmetric geometric shapes; thus, depending on the orientation of the image, one can obtain slightly different measurements when measuring the vertical diameter. The vertical distance measured in the study was best oriented along the long axis of the tooth and perpendicular to the occlusal plane to minimize this error.

The relatively small sample size was a major limitation, as this was a prevalence study; thus, a larger sample size should have strengthened the result. Additionally, accounting for confounding factors such as the presence of occlusal interference and occlusal wear would have strengthened the study.

### Future study

Future investigations comparing the changes in posterior bony defects pre- and postorthodontic treatment [27] can be conducted. Additionally, a study that analyzes bony defect prevalence and size by the buccolingual inclination [12] of individual teeth and/or transverse arch width of study subjects would provide more insight into the interpretations derived from the current study. Another suggestion for a future study is to compare the prevalence of bony defects in different posterior crossbite type subjects by etiology (skeletal crossbite, dental crossbite, functional crossbite) or by the number of teeth and sides involved (single tooth crossbite, segmental crossbite, unilateral crossbite, bilateral crossbite).

Comparisons of the bony defect prevalence between teeth in full crossbite and teeth in edgebite with a larger sample size would be interesting in order to determine if one group shows more bony defects than the other group.

## Conclusion

- Posterior crossbite subjects had a higher prevalence of total bony defects (61.6%) and dehiscence (41.2%), especially buccal dehiscence, than noncrossbite subjects did. This is due to a higher prevalence observed in the noncrossbite teeth in the crossbite group (subgroup 2) than in the noncrossbite teeth in the noncrossbite group (subgroup 3).
- There was no statistically significant difference in the posterior fenestration prevalence between the posterior crossbite subjects and noncrossbite subjects. When divided by subgroup, the noncrossbite teeth in the crossbite group (subgroup 2) showed more fenestration than other subgroups, mainly due to lingual fenestration.
- There was no statistically significant difference in the mean size of dehiscence and fenestration between the posterior crossbite subjects and noncrossbite subjects, as well as among the three subgroups.
- Dehiscences were more prevalent in first premolars than in other posterior teeth.
- Fenestrations were more prevalent in posterior maxillary teeth than in posterior mandibular teeth.

## Data Availability

The datasets used and/or analyzed during the current study are available from the corresponding author on reasonable request.

## Appendix

[Crossbite group consisting of only teeth in full crossbite, excluding edgebite teeth]

When both teeth in crossbite and edgebite were included in the crossbite group, there was a higher prevalence of total bony defects and dehiscence in the crossbite group than in the noncrossbite group, which was statistically significant (Table 4). When only crossbite teeth were included, statistical significance was found in the prevalence of total bony defects, dehiscence, and fenestration as well as the mean size of dehiscence. There was a higher bony defect prevalence and slightly larger mean size of dehiscence (Table 9).

**Table 9:** Prevalence and mean size of total bony defects, dehiscence, and fenestration in the crossbite group (excluding edgebite teeth) and the noncrossbite group

		Crossbite group (1) (excluding edgebite) (*n* = 416) teeth	Noncrossbite group (*n* = 447) teeth	*p* value
Total bony defect prevalence (number of observations)		62.5% (260)	52.1% (233)	0.002**
Dehiscence	Prevalence (number of observations)	41.8% (174)	33.3% (149)	0.010*
	Mean size(mm) ± SD	4.52 ± 2.63	3.96 ± 2.26	0.042*
Fenestration	Prevalence (number of observations)	35.1% (146)	28.6% (128)	0.042*
	Mean size (mm) ± SD	4.36 ± 2.17	4.13 ± 1.83	0.339

**p* < 0.05 statistically significant

***p* < 0.01 statistically highly significant

When the edgebite teeth were excluded from the crossbite group and it was divided into subgroups, the sample size of subgroup 1 was reduced from 110 to 89 (Table 10). The result for bony defect prevalence and mean size comparison was similar to that for the comparison between the original crossbite group and the noncrossbite group (Table 5), since statistically significant differences were found between subgroup 2 and subgroup 3. Table 10 shows a similar trend to Table 4: subgroup 2 had the highest bony defect prevalence and mean size, followed by subgroup 1 and subgroup 3.

**Table 10:** Prevalence and mean size of total bony defects, dehiscence, and fenestration by subgroup. Teeth in edgebite are excluded

		Crossbite group (1) (excluding edgebite) (*n* = 416)		Noncrossbite group (*n* = 447)	*p* value (SG1–SG2)	*p* value (SG1–SG3)	*p* value (SG2–SG3)
		SG1: teeth in crossbite (*n* = 89)	SG2: teeth not in crossbite (*n* = 327)	SG3: teeth not in crossbite (*n* = 447)			
Total bony defect prevalence (number of observations)		59.6% (53)	63.3% (207)	52.1% (233)	0.517	0.200	0.002**
		*p* value = 0.007**					
Dehiscence	Prevalence (number of observations)	39.3% (35)	42.5% (139)	33.3% (149)	0.590	0.277	0.009**
		*p* value = 0.031*					
	Mean size (mm) ± SD	4.47 ± 2.97	4.53 ± 2.55	3.96 ± 2.26	0.132		
Fenestration	Prevalence (number of observations)	32.6% (29)	35.8% (117)	28.6% (128)	0.575	0.455	0.035*
		*p* value = 0.106					
	Mean size (mm) ± SD	4.13 ± 2.20	4.42 ± 2.17	4.13 ± 1.83	0.498		

(Chi-square test for the prevalence comparison, ANOVA for the mean size comparison)

**p* < 0.05 statistically significant

***p* < 0.01 statistically highly significant

For the comparison of the prevalence of dehiscence and fenestration per buccal or lingual side between the crossbite group without edgebite teeth and the noncrossbite group (Table 11), statistical significance was observed only in the prevalence of buccal dehiscence: a higher prevalence was observed in the crossbite group than in the noncrossbite group. This result was similar to that of Table 6.

**Table 11:** Dehiscence and fenestration prevalence and mean size by buccal and lingual surfaces in the crossbite group without teeth in edgebite and the noncrossbite group

		Crossbite group (1) (excluding edgebite) (*n* = 416)	Noncrossbite group (*n* = 447)	*p* value
Buccal dehiscence	Prevalence (number of observations)	32.9% (137)	22.4% (100)	0.001**
	Mean size (mm) ± SD	4.53 ± 2.57	4.27 ± 2.54	0.425
Lingual dehiscence	Prevalence (number of observations)	25.7% (107)	21.35% (95)	0.121
	Mean size (mm) ± SD	3.29 ± 2.09	2.91 ± 1.13	0.106
Buccal fenestration	Prevalence (number of observations)	26.0% (108)	22.8% (102)	0.282
	Mean size (mm) ± SD	4.25 ± 1.90	4.15 ± 1.76	0.676
Lingual fenestration	Prevalence (number of observations)	11.5% (48)	7.8% (35)	0.065
	Mean size (mm) ± SD	4.40 ± 2.59	3.83 ± 1.96	0.280

***p* < 0.01 statistically highly significant

The results for comparison of the prevalence and mean size of the bony defects on the buccal or lingual side among subgroups were similar whether the edgebite teeth were included or excluded in the crossbite group. Statistical significance was found in the prevalence of buccal dehiscence and lingual fenestration between subgroup 2 and subgroup 3 (Table 12).

**Table 12:** Dehiscence and fenestration prevalence and mean size by buccal and lingual surfaces by subgroup. Teeth in edgebite were excluded

		Crossbite group (1) (excluding edgebite) (*n* = 416)		Noncrossbite group (*n* = 447)	*p* value (SG1–SG2)	*p* value (SG1–SG3)	*p* value (SG2–SG3)
		SG1: teeth in crossbite (89)	SG2: teeth not in crossbite (327)	SG3: teeth not in crossbite (447)			
Buccal Dehiscence	Prevalence (number of observations)	29.2% (26)	33.9% (111)	22.4% (100)	0.400	0.164	< 0.001**
	Mean size (mm) ± SD	4.45 ± 2.95	4.56 ± 2.48	4.27 ± 2.54	0.715		
Lingual dehiscence	Prevalence (number of observations)	28.1% (25)	25.1% (82)	21.3% (95)	0.564	0.158	0.211
	Mean size (mm) ± SD	3.61 ± 2.59	3.19 ± 1.92	2.91 ± 1.13	0.165		
Buccal fenestration	Prevalence (number of observations)	28.1% (25)	25.4% (83)	22.8% (102)	0.605	0.286	0.409
	Mean size (mm) ± SD	3.69 ± 1.45	4.42 ± 1.99	4.15 ± 1.76	0.201		
Lingual fenestration	Prevalence (number of observations)	9% (8)	12.2% (40)	7.8% (35)	0.396	0.713	0.041*
	Mean size (mm) ± SD	5.38 ± 3.19	4.21 ± 2.45	3.83 ± 1.96	0.245		

(Chi-square test for the prevalence comparison, ANOVA for the mean size comparison)

**p* < 0.05 statistically significant

***p* < 0.01 statistically highly significant

When comparing the bony defect prevalence between the buccal and lingual sides, the buccal side had a higher prevalence than the lingual side. The difference was larger for the prevalence of fenestration than for the prevalence of dehiscence. This finding was observed regardless of the inclusion or exclusion of edgebite teeth in the crossbite group (Table 8 and table 13).

**Table 13:** Comparison of bony defect prevalence between the buccal and lingual sides. Teeth in edgebite were excluded

			Buccal side only	Lingual side only	Both sides	Buccal total	Lingual total	*p* value
Dehiscence prevalence (number of observations)	Crossbite group (1) (*n* = 416)		16.1% (67)	8.9% (37)	16.8% (70)	29.2% (137)	25.7% (107)	0.022
		SG 1 (*n* = 89)	11.2% (10)	10.1% (9)	18.0% (16)	27.3% (26)	28.1% (25)	0.868
		SG 2 (*n* = 327)	17.4% (57)	8.6% (28)	16.5% (54)	33.9% (111)	25.1% (82)	0.013*
	Noncrossbite group (*n* = 447)	SG 3 (*n* = 447)	12.1% (54)	11.0% (49)	10.3% (46)	22.4% (100)	21.3% (95)	0.686
Fenestration prevalence (number of observations)	Crossbite group (1) (*n* = 416)		23.3% (97)	8.9% (37)	2.6% (11)	26% (108)	11.5% (48)	< 0.001**
		SG 1 (*n* = 89)	23.6% (21)	4.5% (4)	4.5% (4)	28.1% (25)	9.0% (8)	< 0.001**
		SG 2 (*n* = 327)	23.2% (76)	10.1% (33)	2.1% (7)	25.4% (83)	12.2% (40)	< 0.001**
	Noncrossbite group (*n* = 447)	SG 3 (*n* = 447)	20.8% (93)	5.8% (26)	2.0% (9)	22.8% (102)	7.8% (35)	< 0.001**

(*p* values for the prevalence comparison between the buccal total and lingual total)

**p* < 0.05 statistically significant

***p*<0.01 statistically highly significant

## Abbreviations

CBCT: Cone beam computed tomography
CEJ: Cementoenamel junction
FH: Frankfort-Horizontal
FOV: Field of view
ICC: Intraclass correlation coefficient
SG1: Subgroup 1
SG2: Subgroup 2
SG3: Subgroup 3
